# Supplementary material for: Auxotrophic and prototrophic conditional genetic networks reveal the rewiring of transcription factors in Escherichia coli
Source: Nat Commun. 2022 Jul 14;13:4085. doi: 10.1038/s41467-022-31819-x (PMC9283627; doi:10.1038/s41467-022-31819-x)
Supplement: Supplementary file 1 — Supplementary Information [file 41467_2022_31819_MOESM1_ESM.pdf]

Supplementary Information

Auxotrophic and Prototrophic Conditional Genetic Networks Reveal the Rewiring of Transcription Factors in *Escherichia coli*

Alla Gagarinova[1,2], Ali Hosseinnia[1,2], Matineh Rahmatbakhsh[1,2], Zoe Istace[1], Sadhna Phanse[1], Mohamed Taha Moutaoufik[1], Mara Zilocchi[1], Qingzhou Zhang[1], Hiroyuki Aoki[1], Matthew Jessulat[1], Sunyoung Kim[1], Khaled A. Aly[1], and Mohan Babu[1,§]

[1]Department of Biochemistry, University of Regina, Regina, Saskatchewan, Canada

[2]These authors contributed equally: Alla Gagarinova, Ali Hosseinnia, Matineh Rahmatbakhsh

[§]Correspondence: mohan.babu@uregina.ca

<u>Contents</u>:

1. Supplementary Figures: 1-7

2. Supplementary Data: 1-17

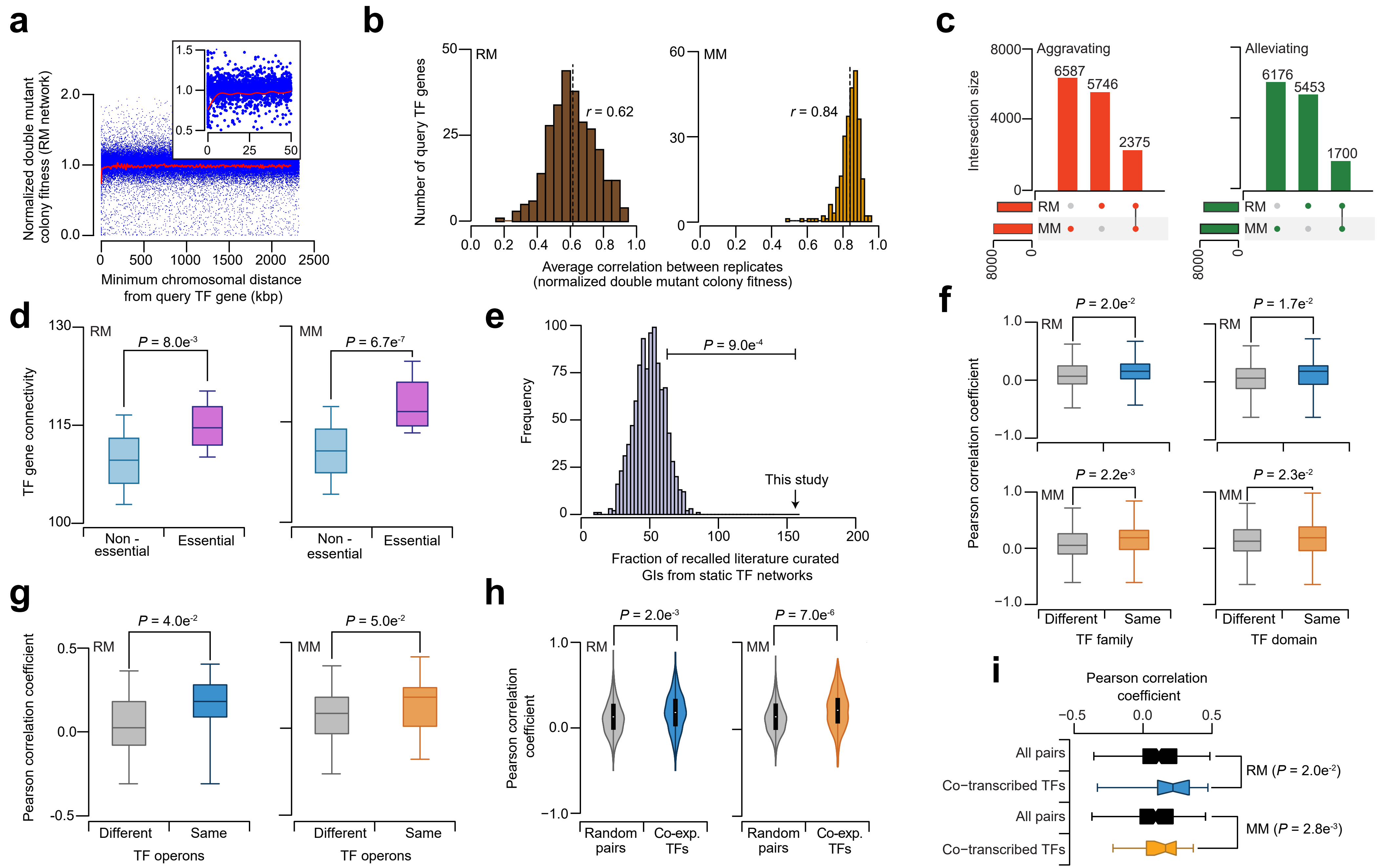

Supplementary Fig. 1 Quality control analyses in RM and MM networks. a Normalized colony growth fitness of all double mutants from RM network is plotted against the shortest chromosomal distance from TF donor mutant strain. Double mutants with low recombination frequency due to linkage suppression within 30 kbp from the query loci are shown in inset. **b** Average correlation of normalized colony growth fitness of all double mutant gene pairs is shown for each query 'TF' gene mutant in replicate RM or MM genetic screens. Dotted line is average correlation. **c** Overlap of GIs between RM and MM networks. **d** Non-essential and essential TF gene pair connectivity in RM ($P = 8.0e^{-3}$) and MM ($P = 6.7e^{-7}$) networks. **e** Agreement of literature curated GIs from this study vs. the frequencies of expected GI pairs by random sampling (blue distribution with 1000 null models) in static networks. $P = 9.0e^{-4}$ by permutation test. **f-i** GI correlation profiles of genes within the same transcriptional regulatory family (**f**; RM: $P = 2.0e^{-2}$ and MM: $P = 2.2e^{-3}$), domain (**f**; RM: $P = 1.7e^{-2}$ and MM: $P = 2.3e^{-2}$) and TF operons (**g**, RM: $P = 4.0e^{-2}$ and MM: $P = 5.0e^{-2}$), as well as co-expressed (**h**; Co-exp; RM: $P = 2.0e^{-3}$ and MM: $P = 7.0e^{-6}$) or co-transcribed TFs (**i**; RM: $P = 2.0e^{-2}$ and MM: $P = 2.8e^{-3}$) compared to gene pairs drawn from different family, domains, operons or random gene pairs. *P*-value by two-sided Wilcoxon signed-rank (**d, f-i**) test. Box plot from left to right for panels **d, f-h** and from top to bottom for panel **i** are shown for RM with maximum (116 and 120; 0.6 and 0.7; 0.6 and 0.7; 0.39 and 0.40; 0.9 and 0.9; 0.49 and 0.48), minimum (103 and 110; -0.5 and -0.4; -0.6 and -0.6; -0.3 and -0.3; -0.5 and -0.5; -0.38 and -0.36), quartile 1 (Q1; 106 and 112; -0.1 and 0.0; -0.2 and -0.1; -0.1 and 0.1; 0.0 and 0.01; -0.01 and 0.1), Q2 (109 and 115; 0.1 and 0.2; 0.0 and 0.2; 0.0 and 0.19; 0.12 and 0.12; 0.11 and 0.21), and Q3 (113 and 118; 0.2 and 0.3; 0.2 and 0.3; 0.2 and 0.4; 0.13 and 0.132; 0.25 and 0.32). Likewise, box plot from left to right for panels **d, f-h** and from top to bottom for panel **i** are shown for MM with maximum (118 and 125; 0.6 and 0.7; 0.7 and 0.9; 0.40 and 0.45; 0.8 and 0.8; 0.45 and 0.38), minimum (104 and 114; -0.7 and -0.7; -0.7 and -0.7; -0.25 and -0.17; -0.48 and -0.48; -0.39 and -0.21), Q1 (108 and 115; -0.2 and -0.1; -0.1 and -0.1; -0.01 and 0.12; 0.0 and 0.02; -0.02 and 0.01), Q2 (117 and 177; 0.0 and 0.1; 0.0 and 0.1; 0.1 and 0.2; 0.12 and 0.22; 0.10 and 0.17), and Q3 (114 and 122; 0.2 and 0.2; 0.2 and 0.3; 0.2 and 0.3; 0.13 and 0.14; 0.21 and 0.24). Source data are provided as a Source Data file.

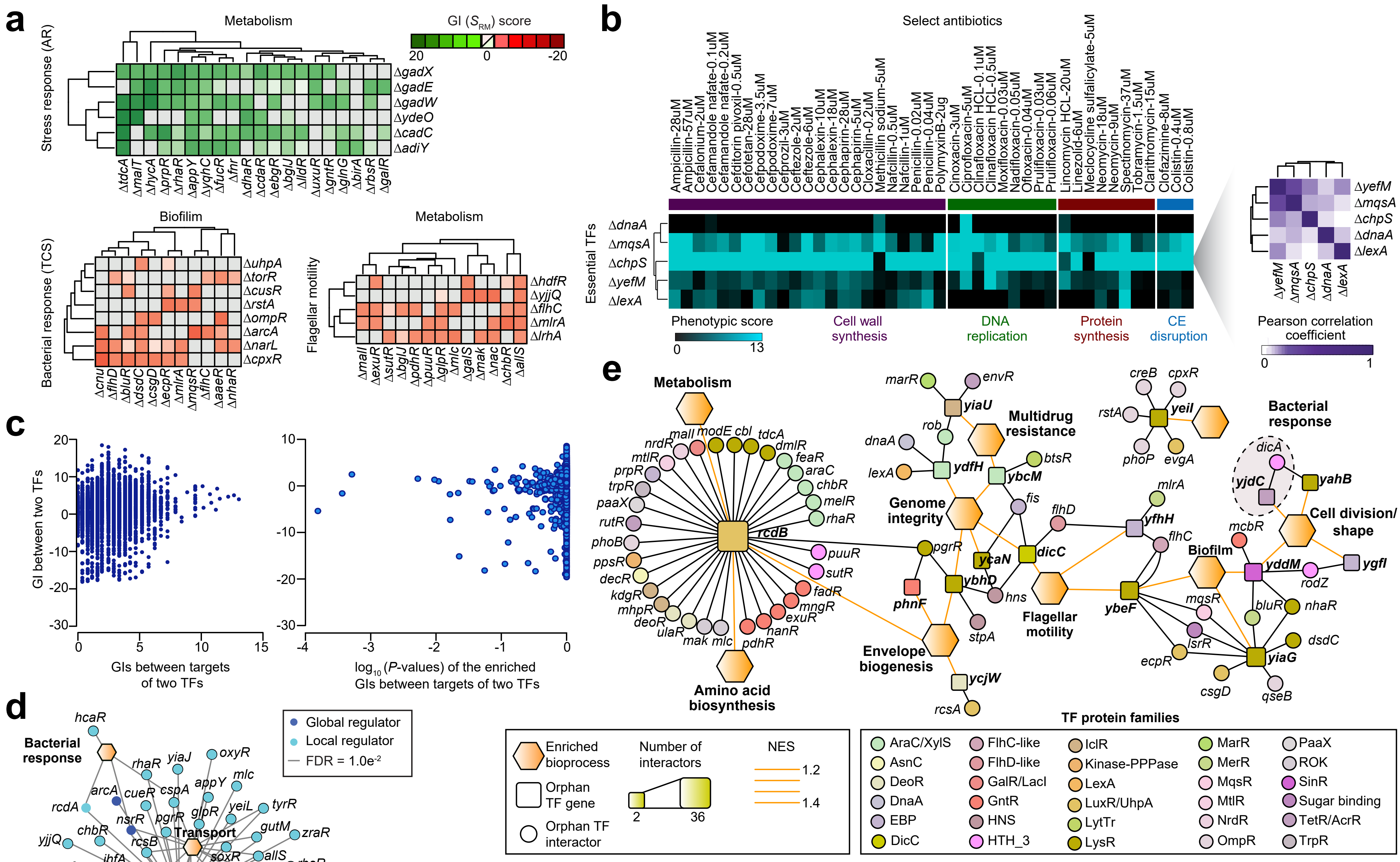

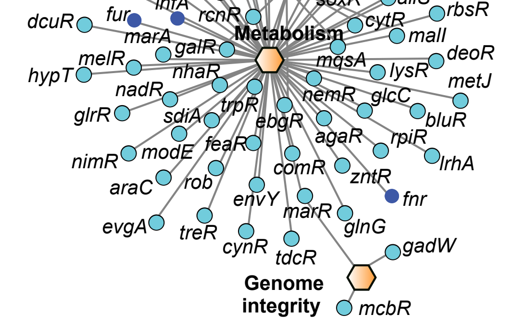

Supplementary Fig. 2 TF epistatic connectivity from static RM network in enriched bioprocesses. a Functional crosstalk or unexpected functional dependencies of TFs in the indicated functional categories. AR, acid resistance; TCS, two-component system. **b** Antitoxin (*chpS*, *mqsA*, *yefM*) and SOS regulon (*dnaA*, *lexA*) single gene deletion mutant resistance profiles (represented as phenotypic score) to the antibiotics targeting various bioprocesses at indicated concentrations from our ongoing essential phenomics screens is shown along with their correlated GI profiles. The phenotypic fitness score was generated as we previously described (Kintses et al., 2019 *Nature Communications* 10, 5731); CE, cell envelope. **c** The GIs between two TFs is plotted against the number of GIs between targets of two TFs (left; n = 15,274 gene pairs) and the P-values of enrichments of GIs between their targets of two TFs (n = 15,274 gene pairs). **d, e** GIs involving global or local TF regulators (**d**; **Supplementary Data 5**; FDR ≈ 1%) or GIs between an orphan and annotated gene of two different TF families (**e**) displaying significant enrichment in the indicated bioprocesses; NES, normalized enrichment score. P-value by one-tailed hypergeometric (**c, d**) and two-tailed Kolmogorov–Smirnov (**e**) tests. P-value adjusted using the Benjamini-Hochberg FDR correction for panels **d** and **e**. Source data are provided as a Source Data file.

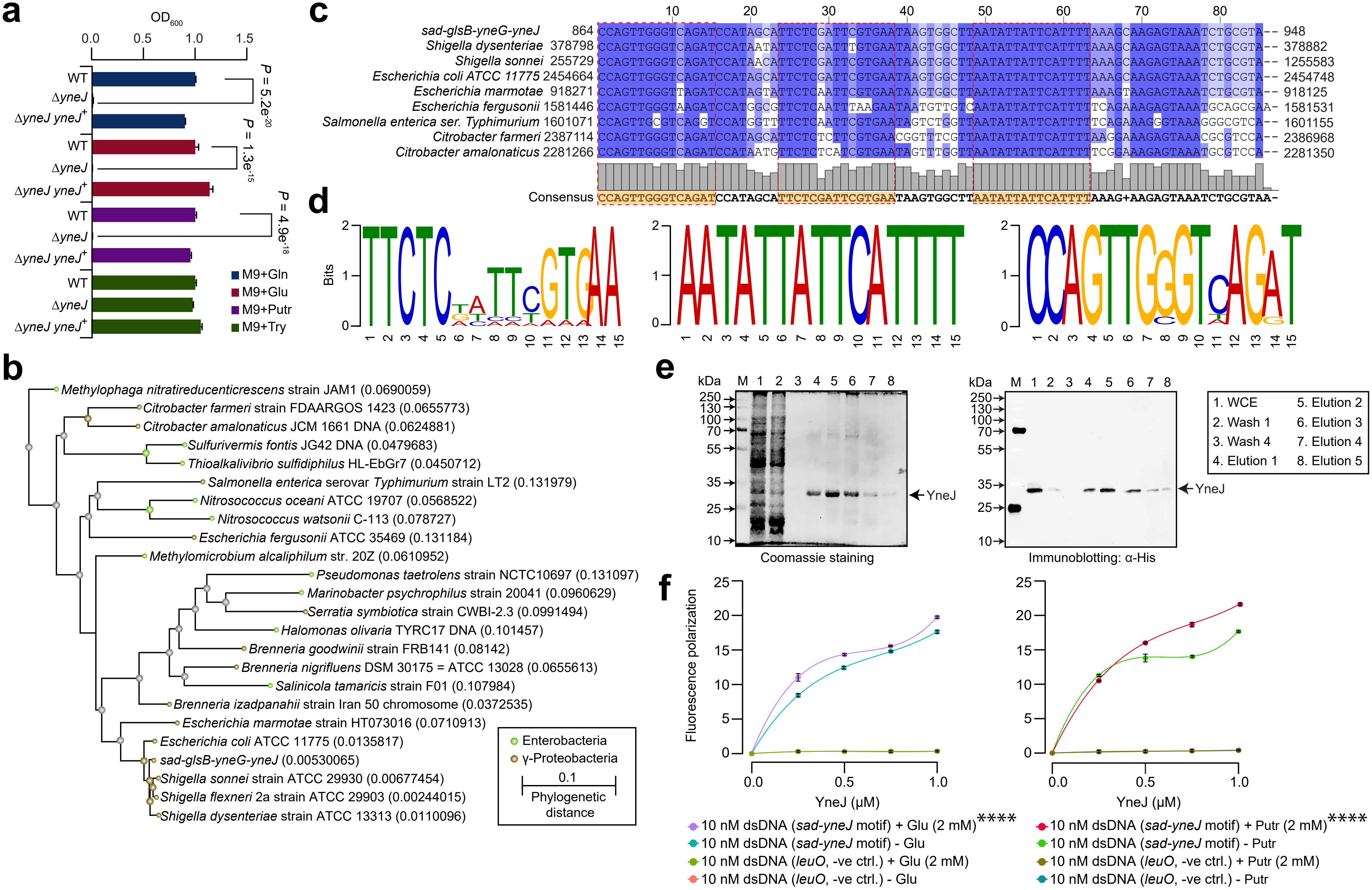

Supplementary Fig. 3 Conservation analysis and functional assays of YneJ. a OD_{600} growth rate readings ($n = 12$ biologically independent experiments) of wild-type (WT), *yneJ* mutant, and *yneJ* overexpression (+) in *yneJ* mutant strain measured at 24-hr time point in M9 carbon source-limiting medium containing Gln (0.5% Glutamine), Glu (0.5% Glutamate), Putr (0.4% Putrescine) or Try (1% Tryptone) as a sole carbon source (instead of glucose), and NH_4Cl as a nitrogen source; significance ($P = 5.2e^{-20}$, $P = 1.3e^{-15}$ and $P = 4.9e^{-18}$) by Student's two-sided *t*-test. **b** Sequence co-conservation of *sad-glsB-yneG* (i.e., *yneI* operon) and *yneJ* in closely related γ-proteobacteria class is shown along with their phylogenetic distance in parenthesis. **c, d** DNA sequence alignment of *yneI* operon and *yneJ* to the indicated γ-Proteobacterial species (**c**) and a consensus motif (**d**) predicted by MEME suite. **e** Coomassie blue staining (left) and immunoblotting (right) of the purified YneJ-His_6-tagged recombinant protein probed with anti-His antibody (1: 1000 dilution) in the whole cell extract (WCE, Lane 1); wash fractions (i.e., eluates from wash fractions of the first and fourth washes with washing buffer, Lanes 2-3), and elution fractions (i.e., eluates eluted five times with 2 ml of elution buffer per elution, Lanes 4-8). This experiment was performed only once to obtain enough YneJ purified recombinant protein. **f** Fluorescence polarization ($n = 4$ biologically independent experiments) at increasing concentration of YneJ protein with 10 nM fluorescence-labeled double-stranded DNA (i.e., fragment from *sad-yneJ* binding site, or promoter region of *leuO* gene that serve as negative control), and in the presence or absence of Glu or Putr (2 mM). Significance (****$P = 5.8e^{-5}$ for Glu and $P = 5.6e^{-8}$ for Putr) calculated using Student's two-sided *t*-test between *sad-yneJ* consensus motif without vs. with Glu or Putr at 1 μM concentration of YneJ protein. Data (**a, f**) are presented as mean ± standard deviation from the indicated number of independent samples. Source data are provided as a Source Data file.

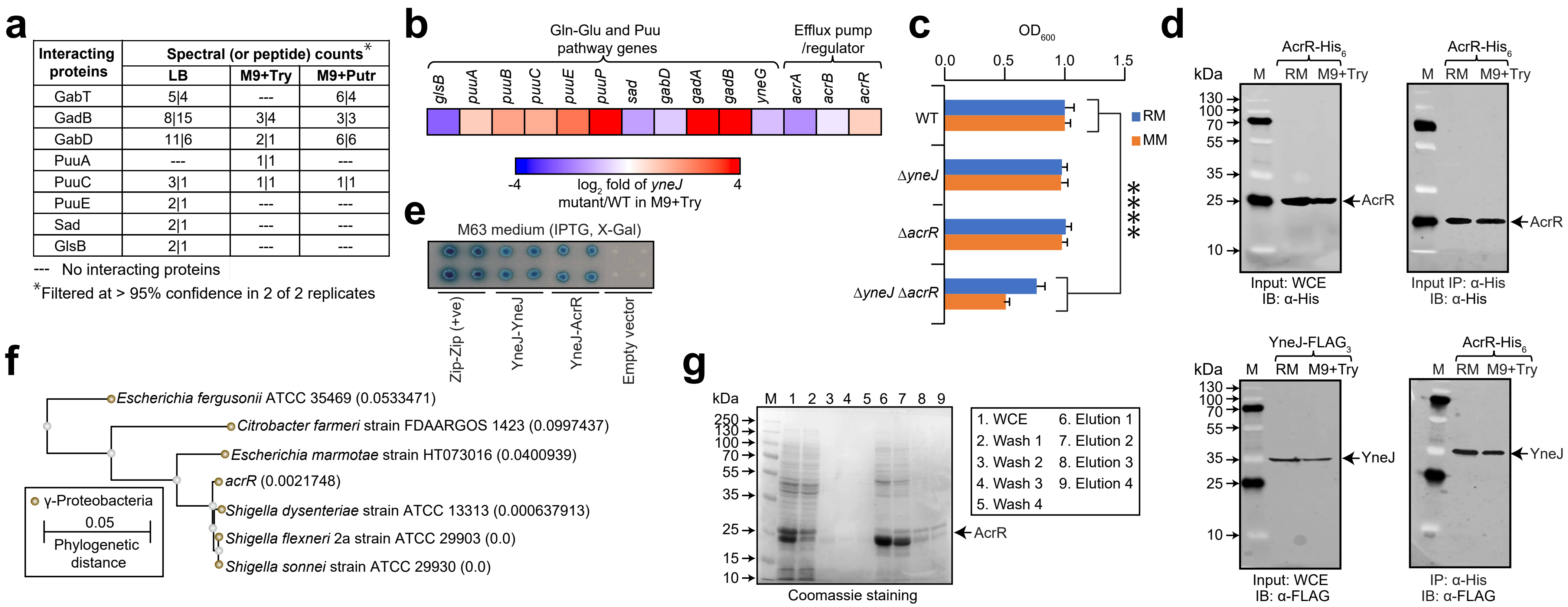

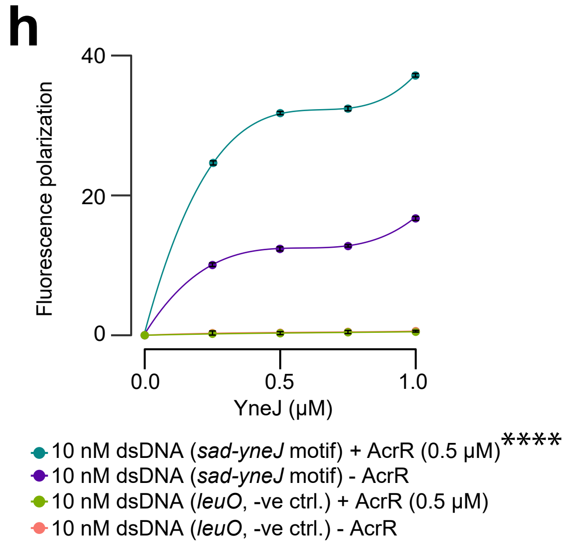

Supplementary Fig. 4 Conservation analysis and functional assays of YneJ and AcrR. a YneJ interaction with indicated proteins by affinity purification and mass spectrometry ($n = 2$ biologically independent experiments) filtered at 95% or greater probability score should present in 2 of 2 replicates in different growth conditions tested; Luria-Bertani (LB), M9 medium containing putrescine (0.4% Putr) or tryptone (1% Try). **b** Transcript levels of indicated genes measured by bulk RNA-seq in *yneJ* mutant vs. wild-type (WT) in 1% M9+Try at 32°C are shown as $\log_2$fold change. **c** Growth rates ($n = 12$ biologically independent experiments) of WT and indicated mutant strains in RM or MM growth conditions at 32°C over 24 hrs. Significance (****$P = 9.2e^{-18}$ for RM and $P = 7.0e^{-26}$ for MM) between WT and Δ*yneJ* Δ*acrR* mutant strains was computed by Student's two-sided *t*-test. **d** Immunoprecipitates (IP) and the input IP or whole cell extracts (WCE) of the affinity-tagged proteins from the indicated growth conditions immunoblotted (IB) with anti-His (1:1000 dilution) or -FLAG (1:5000 dilution) antibody; molecular masses (kDa) of marker proteins are indicated. Immunoblot is representative of 2 independent biological experiments. **e** Confirmation of YneJ-AcrR interaction by bacterial two-hybrid. Plasmids pUT18C-Zip and pKT25-Zip containing DNA sequence coding for leucine zipper motif was used as a positive control. Blue growth, strong interaction; white growth, no interaction. Bacterial two-hybrid is representative of 3 independent biological experiments. **f** Sequence co-conservation of *acrR* in γ-proteobacteria class of the Enterobacteriaceae family is shown along with their phylogenetic distance in parenthesis. **g** Coomassie blue staining of the purified AcrR-His_6-tagged recombinant protein in the: whole cell extract (WCE, Lane 1); wash fractions (i.e., eluates collected from wash fractions of the first, second, third and fourth washes with washing buffer, Lanes 2-5), as well as elution fractions (i.e., eluates eluted four times with 2 ml of elution buffer per elution, Lanes 6-9). This experiment was performed only once to obtain enough AcrR purified recombinant protein. **h** Fluorescence polarization ($n = 4$ biologically independent experiments) measured at increasing YneJ recombinant protein concentration with 10 nM fluorescence-labeled double-stranded DNA (i.e., fragment from *sad-yneJ* binding site, or promoter region of *leuO* gene that serve as negative control), and in the presence or absence of purified AcrR recombinant protein (0.5 μM). Significance (****$P = 6.9e^{-11}$) by Student's two-sided *t*-test between *sad-yneJ* consensus motif with AcrR vs. without AcrR at 1 μM concentration of YneJ protein. Data (**c, h**) are presented as mean ± standard deviation from the indicated number of independent samples. Source data are provided as a Source Data file.

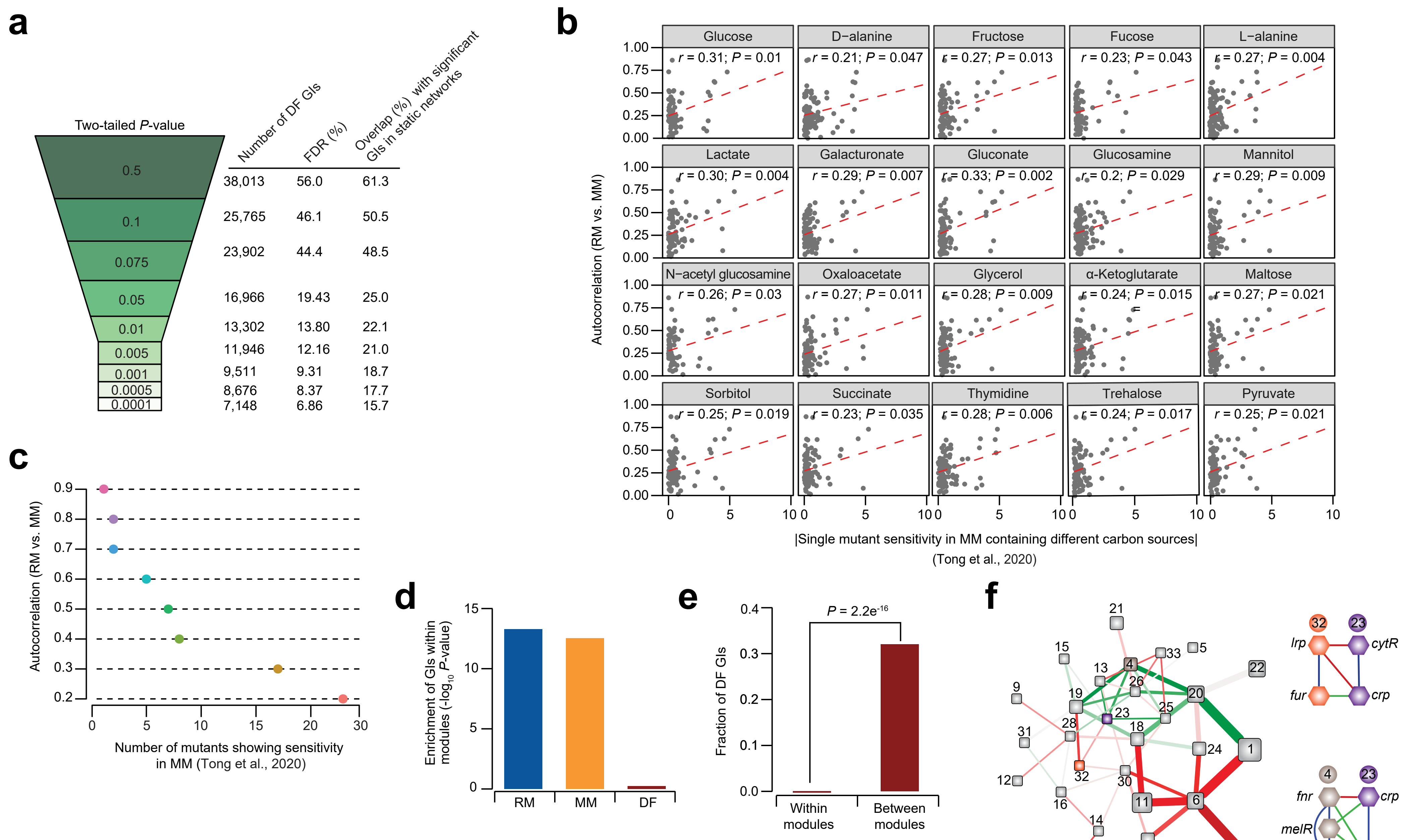

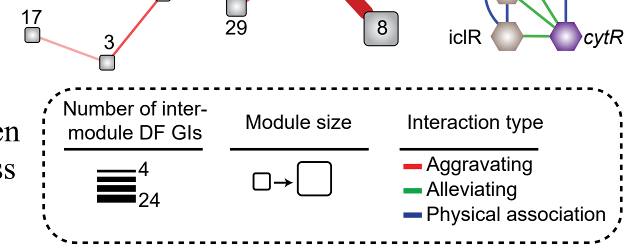

Supplementary Fig. 5 Static and/or differential network analyses. a Number of differential (DF) GIs with increasing levels of significance and their percentage overlap with static (RM or MM) networks. The false discovery rate (FDR) for the corresponding *P*-values is adjusted using the Benjamini-Hochberg correction, and calculated based on the number of GIs expected at random. **b, c** Correlation of GI profiles of each TF gene between RM and MM static networks (i.e., Autocorrelation) is plotted against single TF gene deletion mutant growth fitness sensitivity in MM containing with (**b**) or without (**c**) different carbon sources from the phenotypic genetic screen (Tong et al., 2020). **d** Enrichment of GIs from static RM (n = 35 out of 2,346 gene pairs) or MM (n = 34 of 2,346) and DF (n = 5 of 2,346) networks within modules. **e** Fraction of DF GI gene pairs (n= 1,647 tested) enriched for between (n = 528 observed, P =$2.2e^{-16}$) than within (n = 5 observed) modules. **f** Enrichment of functional modules connected by DF GIs (see **Supplementary Data 11**) is shown along with a select set of inter-module interactions in periphery. *P*-value by two-sided Fisher's Exact (**d, e**), and Z-score permutation (**f**) tests. Source data are provided as a Source Data file.

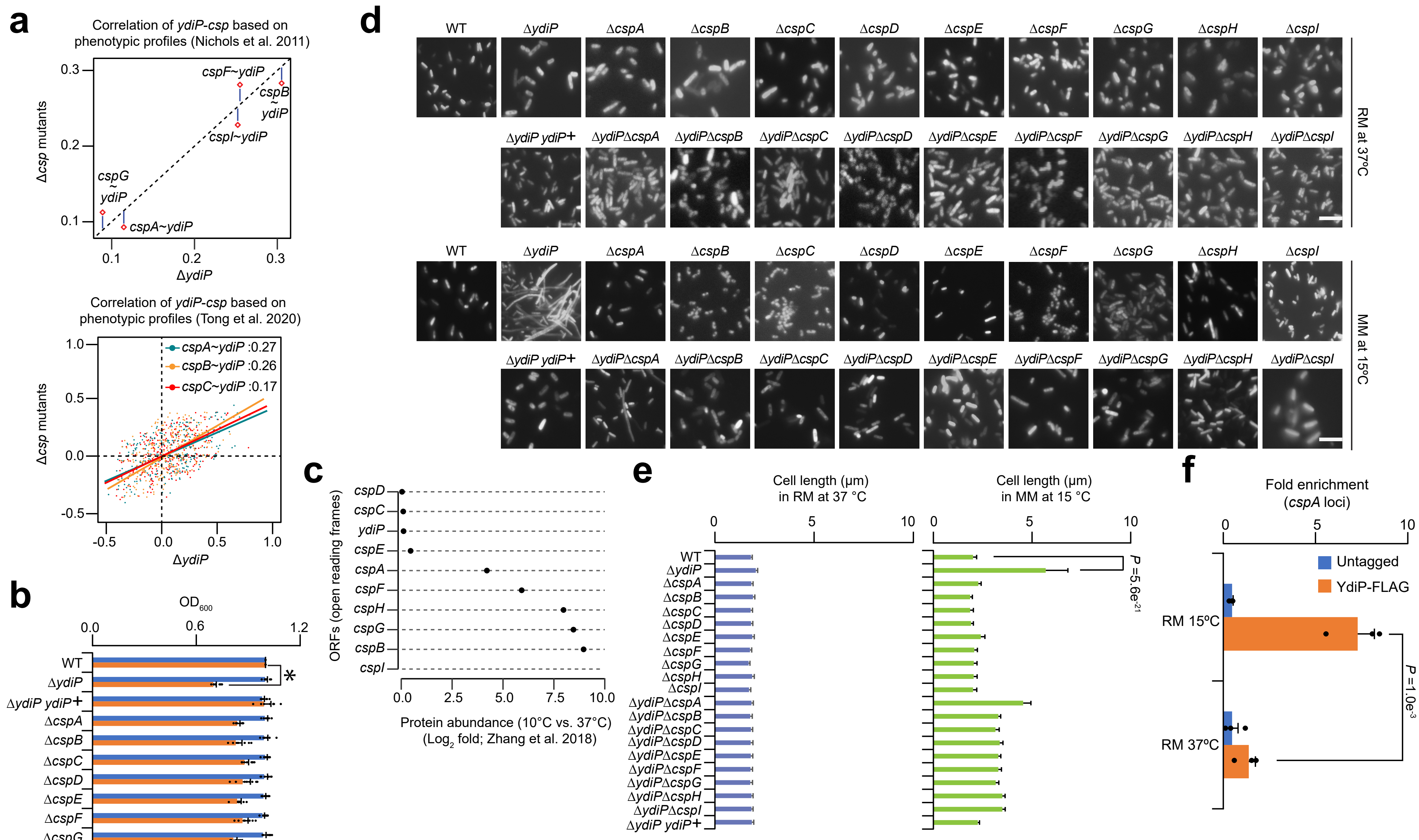

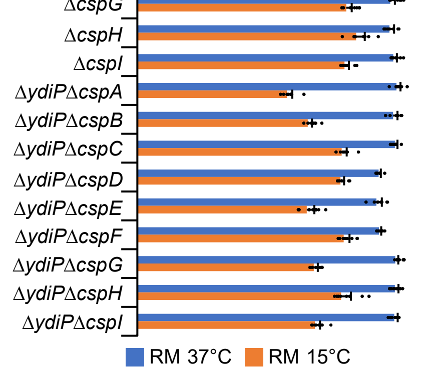

Supplementary Fig. 6 YdiP association with cold shock proteins. a Positively correlated phenotypic profiles between *ydiP* and indicated *csp* single mutant strains in the large-scale phenomics screens performed under a variety of drug treatments (Nichols et al., 2011) and carbon environments (n = 561 GIs tested between each of the indicated *csp* and *ydiP* gene; Tong et al., 2020). **b** OD_{600} growth rate readings (n = 8 biologically independent experiments; *P = 5.4e^{-17} by Student's two-sided t-test) of wild-type (WT) and indicated *ydiP* and *csp* single and double mutants, and *ydiP* overexpression (+) in *ydiP* deletion mutant strain measured at 24-hr time point in RM growth condition upon a temperature shift from 37°C to 15°C. **c** Protein abundance of *ydiP* and the indicated *csp* ORFs from the bacterial ribosome profiling measured at 6 hr after cold shock of 10°C in MOPS rich defined media (minus methionine) compared to before cold shock at 37°C (Zhang et al., 2018). **d, e** Representative cell morphology micrographs (**d**) of *ydiP* and *csp* single and double mutants, as well as overexpression ('+') of *ydiP* in *ydiP* mutant strain in RM at 37°C and MM at 15°C are shown along with cell length measurements (**e**; $n \geq 25$ cells over five biologically independent experiments; P = 5.6e^{-21} by Student's two-sided t-test); Scale bar, 10 μm. **f** ChIP-qPCR (n = 3 biologically independent experiments; P = 1.0e^{-3} by Student's two-sided t-test) showing the fold enrichment of YdiP binding site to *cspA* using a chromosomal YdiP $FLAG_3$-tagged strain vs. an untagged strain after normalizing to a negative control (i.e., primers designed away from *cspE* was amplified from YdiP-FLAG) in RM at different temperature conditions. Data (**b, e, f**) are presented as mean ± standard deviation from the indicated number of independent samples. Source data are provided as a Source Data file.

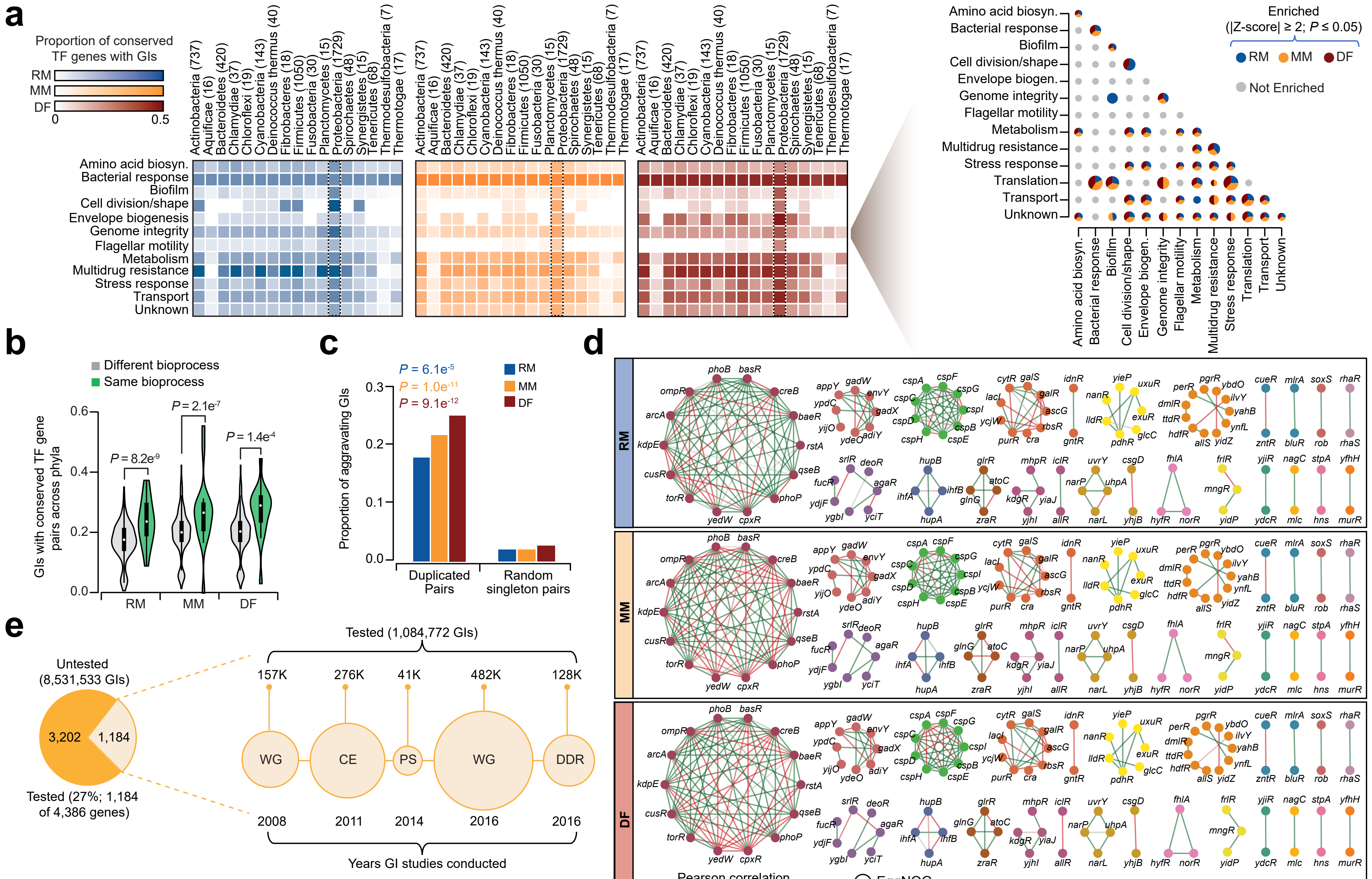

Supplementary Fig. 7 Conserved TF GIs across species and bacterial TF paralogs in static and DF networks. a Heatmap of conserved static and differential (DF) GIs based on co-occurrence of TF orthologs in the indicated bioprocesses across bacterial phyla. Number of genomes sequenced within each phylum is shown in parentheses. Conserved interprocess gene pairs connected by GIs in static and DF networks for γ-Proteobacteria (vertical dotted line) is shown as a zoom-in. Node size is proportional to the number of GIs conserved within the γ-Proteobacterial species. *P*-value by Z-score permutation test. **b** Proportion of GIs with conserved TF pairs in the same (n = 17 gene pairs) or different (n = 153 TF pairs) bioprocess across phyla in static (RM: n = 15,274 GI pairs tested, P = 8.2e^{-9}; MM: n = 16,836, P = 2.1e^{-7}) or DF (n = 16,966, P = 1.4e^{-4}) networks. *P*-value by two-sided Wilcoxon signed-rank test. Violin plot from left to right is shown for RM, MM and DF networks with maximum (0.35 and 0.37; 0.42 and 0.30; 0.45 and 0.44), minimum (0.01 and 0.09; 0.01 and 0; 0.04 and 0.03), quartile 1 (Q1; 0.14 and 0.19; 0.16 and 0.21; 0.18 and 0.23), Q2 (0.17 and 0.23; 0.19 and 0.27; 0.20 and 0.29) and Q3 (0.20 and 0.30; 0.23 and 0.30; 0.24 and 0.32). **c** Proportion of aggravating GIs among duplicate pairs vs. random singleton pairs (RM: n = 37 out of 8,128 tested gene pairs; MM: n = 46 out of 8,962; DF: n = 51 out of 9,208). *P*-value (RM: P = 6.1e^{-5}, MM: P = 1.0e^{-11}; DF: P = 9.1e^{-12}) by two-sided Fisher's Exact test. **d** Correlated GI patterns among TF paralog pairs in the static and DF networks. **e** Summary of the current GI status in *E. coli* for over one-fourth (27%; 1,184 of 4,386) of its genome, focusing on various cellular processes such as whole-genome (WG), cell envelope (CE), protein synthesis (PS), and DNA damage response (DDR). Source data are provided as a Source Data file.
