# Supplementary material for: Auxotrophic and prototrophic conditional genetic networks reveal the rewiring of transcription factors in Escherichia coli
Source: Nat Commun. 2022 Jul 14;13:4085. doi: 10.1038/s41467-022-31819-x (PMC9283627; doi:10.1038/s41467-022-31819-x)
Supplement: Supplementary file 2 — Reporting Summary [file 41467_2022_31819_MOESM2_ESM.pdf]

nature portfolio

Corresponding author(s): Babu, Mohan

Last updated by author(s): Jun 27, 2022

Reporting Summary

Nature Portfolio wishes to improve the reproducibility of the work that we publish. This form provides structure for consistency and transparency in reporting. For further information on Nature Portfolio policies, see our Editorial Policies and the Editorial Policy Checklist.

Statistics

For all statistical analyses, confirm that the following items are present in the figure legend, table legend, main text, or Methods section.

n/a	Confirmed	
☐	☒	The exact sample size (*n*) for each experimental group/condition, given as a discrete number and unit of measurement
☐	☒	A statement on whether measurements were taken from distinct samples or whether the same sample was measured repeatedly
☐	☒	The statistical test(s) used AND whether they are one- or two-sided<br>*Only common tests should be described solely by name; describe more complex techniques in the Methods section.*
☒	☐	A description of all covariates tested
☒	☐	A description of any assumptions or corrections, such as tests of normality and adjustment for multiple comparisons
☐	☒	A full description of the statistical parameters including central tendency (e.g. means) or other basic estimates (e.g. regression coefficient) AND variation (e.g. standard deviation) or associated estimates of uncertainty (e.g. confidence intervals)
☐	☒	For null hypothesis testing, the test statistic (e.g. *F*, *t*, *r*) with confidence intervals, effect sizes, degrees of freedom and *P* value noted<br>*Give P values as exact values whenever suitable.*
☒	☐	For Bayesian analysis, information on the choice of priors and Markov chain Monte Carlo settings
☒	☐	For hierarchical and complex designs, identification of the appropriate level for tests and full reporting of outcomes
☐	☒	Estimates of effect sizes (e.g. Cohen's *d*, Pearson's *r*), indicating how they were calculated

Our web collection on statistics for biologists contains articles on many of the points above.

Software and code

Policy information about availability of computer code

Data collection	ZEN 3.5 blue edition and ImageJ (ver. 1.53q) software was used for imaging. LightCycler® Software (ver. 4.1) was used to analyze the real-time PCR quantitative data generated from the Roche LightCycler® 96 Instrument.
Data analysis	1. Bioconductor MOSAiCS (MOdel-based one and two Sample Analysis and Inference for ChIP-Seq; ver. 2.9.0) package for peak calling.<br>2. Raw ChIP-sequence reads were mapped to the Escherichia coli W3110 chromosome using bowtie2 (ver. 2.4.0) open-source software.<br>3. Using Salmon algorithm (ver. 1.4.0), the sequence reads were mapped to the E. coli W3110 reference genome, and subsequently performed an alignment-free based quantification to determine the transcript abundance.<br>4. Mass spectra were mapped to the reference W3100 E. coli protein sequences using SEQUEST (ver. 27 - rev.9) and STATQUEST (ver. 10) probability algorithms.<br>5. Cell length measured using the ImageJ plugin (ver. 1.53q).<br>6. Highly conserved sequences in Proteobacterial species were determined using Clustal Omega (ClustalO) multiple sequence alignment.<br>7. BLAST Tree View Widget was used to cluster sequences according to distances from query sequence for constructing a phylogenetic tree.<br>8. Static and differential genetic interaction score was generated using multiplicative, or machine learning-based Gaussian models.<br>9. Data analysis was performed using Windows Excel version within Microsoft Office 365, and R software package (ver. 4.2.0).

For manuscripts utilizing custom algorithms or software that are central to the research but not yet described in published literature, software must be made available to editors and reviewers. We strongly encourage code deposition in a community repository (e.g. GitHub). See the Nature Portfolio guidelines for submitting code & software for further information.

Data

Policy information about availability of data

All manuscripts must include a data availability statement. This statement should provide the following information, where applicable:

- Accession codes, unique identifiers, or web links for publicly available datasets
- A description of any restrictions on data availability
- For clinical datasets or third party data, please ensure that the statement adheres to our policy

1. Data that support the findings of this study is available at our resource website (http://ecoli.med.utoronto.ca/eMap/TF). ChIP-seq data have been deposited in NCBI under the BioProject accession number PRJNA771186, and are publicly available. All source data are provided in the Source Data file. These information has been included under the 'Data Availability Statement' in the manuscript.
2. Paralogous transcription factor genes passing the set criteria were assigned to bacterial non-supervised orthologous groups (BactNOGs) predicted by EggNOG database.
3. Gene sequences from prokaryotic species were retrieved from NCBI non-redundant protein database to perform conservation and phylogenetic analyses.

Field-specific reporting

Please select the one below that is the best fit for your research. If you are not sure, read the appropriate sections before making your selection.

☒ Life sciences ☐ Behavioural & social sciences ☐ Ecological, evolutionary & environmental sciences

For a reference copy of the document with all sections, see nature.com/documents/nr-reporting-summary-flat.pdf

Life sciences study design

All studies must disclose on these points even when the disclosure is negative.

Sample size	Sample size (n= 3 or n ≥10) from each biologically independent experiment and their associated statistical significance was indicated in the figure legends. Based on our experience and previously published studies, sufficient sample size was chosen to yield enough statistical power to derive biological conclusions.
Data exclusions	No data were excluded from the analysis, except in Fig. 3i where one of the value from the three replicates of WT YjdC+ was excluded as it was deemed as an outlier. We have highlighted this outlier in the source data file under the sheet "Fig 3i".
Replication	Experiments were performed with a minimum of three independent biological replicates, and all attempts at replication were successful.
Randomization	In the case of module crosstalk analysis, each module membership of the target genes was randomly reassigned for 1,000 interactions, and the resulting inter-module genetic interaction distribution from randomized network(s) was transformed to a Z-score. Next, while comparing static transcription factor networks to previously reported genetic interactions, we used random sampling to derive statistical significance. Likewise, genetically interacting transcription factor genes present within the same functional domain or transcriptional regulatory family, and within the same operon in E. coli that had more positively correlated genetic profiles as pairs of co-expressed or co-transcribed transcription factors were compared to random gene pairs. Further, genetic interactions among putative paralogous gene pairs, comprising transcription factor genes, that tend to be more common in static and differential networks was compared to random singleton gene pairs to conclude that genetic redundancy between transcription factor duplicates is modulated in a dynamic manner under environmental stress conditions.
Blinding	There is no blinding employed with respect to the sample selection due to the use of objective means of quantification. Nevertheless, some of the critical follow-up experiments and genetic screens during data collection were performed in a blinded manner. In the latter, strains were randomized on plates and codes were used instead of informative strain identifiers until all the screens and analyses were completed.

Reporting for specific materials, systems and methods

We require information from authors about some types of materials, experimental systems and methods used in many studies. Here, indicate whether each material, system or method listed is relevant to your study. If you are not sure if a list item applies to your research, read the appropriate section before selecting a response.

Materials & experimental systems

n/a	Involved in the study	
☐	☒	Antibodies
☒	☐	Eukaryotic cell lines
☒	☐	Palaeontology and archaeology
☒	☐	Animals and other organisms
☒	☐	Human research participants
☒	☐	Clinical data
☒	☐	Dual use research of concern

Methods

n/a	Involved in the study	
☐	☒	ChIP-seq
☒	☐	Flow cytometry
☒	☐	MRI-based neuroimaging

Antibodies

Antibodies used	1. Input' and 'mock' ChIP experiments was performed using anti-FLAG as well as rabbit and mouse immunoglobulin G antibody. The catalog number, supplier name, and other details are shown below.<br><br>2. Co-immunoprecipitation and immunoblotting experiments were performed using the commercial anti-FLAG and anti-His antibodies (see below), while GroEL (HSP60) antibody was obtained from Dr. Walid Houry lab at the University of Toronto. We used 1:15000 dilution for HSP60.<br><br>3. Antibodies used in Co-immunoprecipitation:<br><br>a. Monoclonal Anti-Flag M2 antibody in mouse<br>Supplier: Sigma<br>Cat#: F1804<br>Lot#: SLBN5629V<br>Experiment: Co-immunoprecipitation, ChIP-seq or ChIP-qPCR<br>Dilution: 1:5000<br><br>b. Anti-His-Tag Antibody (H-3) antibody in mouse<br>Supplier:Santa Cruz<br>Cat#: Sc-8036<br>Lot#: CO-118<br>Experiment: Co-immunoprecipitation<br>Dilution: 1:1000<br><br>c. Anti-Mouse IgG (whole molecule)-peroxidase antibody produced in goat<br>Supplier: Sigma<br>Cat#: A4416-1ML<br>Lot#: SLCD0197<br>Experiment: Co-immunoprecipitation<br>Dilution: 1:2000<br><br>d. Rabbit IgG Isotype Control<br>Supplier: Invitrogen<br>Cat#: 10500C<br>Lot#: TA262556<br>Experiment: ChIP-seq (Input)<br>Dilution: 1:80<br><br>e. IgG from mouse serum<br>Supplier: Sigma<br>Cat#: 15381<br>Lot#: SLBQ7097V<br>Experiment: ChIP-seq (Mock)<br>Dilution: 1:135
Validation	The antibodies validated by the manufacturer, as well as in this study are listed below:<br>a. Monoclonal Anti-Flag M2 antibody in mouse: The Anti-FLAG M2 mouse, affinity purified monoclonal antibody is validated by the manufacturer through immunoblotting from mammalian crude cell lysates, as well as verified in previous publication (Nature Communications 2015, 6, 6253).<br>b. Anti-His-Tag Antibody (H-3) antibody in mouse: The affinity purified anti-His-Tag moue monoclonal antibody is raised against a His-tagged recombinant protein and validated by the manufacturer through immunoblotting of phosphatase-treated and untreated His-tagged active human recombinant p70 S6 kinase. This antibody has also been validated in many previous publications, including in Nature Communications 2022, 13, 1585.<br>c. Anti-Mouse IgG (whole molecule)-peroxidase antibody produced in goat: Anti-Mouse IgG (whole molecule)-Peroxidase antibody is purified and specific for mouse IgGs and is validated by the manufacturer using ELISA. As well, the antibody has been validated in previous publication (The Journal of Cell Biology 2011, 192, 1005-21).<br>d. Rabbit IgG Isotype Control: The purified isotype control rabbit IgG that is validated in a previous publication (Cell Reports 2022, 38, 110534) has been shown on the manufactures website.<br>e. IgG from mouse serum: Mouse IgG purified from normal mouse serum by fractionation and ion-exchange chromatography that is validated in a previous publication (Molecular Neurobiology 2016, 54, 4921-4935) has been shown on the manufactures website.<br>f. The affinity purified GroEL (HSP60) antibody that has been validated by Walid Houry's lab (University of Toronto) in the GroEL immunoprecipitates of Escherichia coli cell lysates by immunoblotting is shown in one of their previous publication (Cell 1997, 90, 491-500), and our study in Fig. 6e also confirms the antibody specificity in E. coli cell lysates at different temperatures over time.

ChIP-seq

Data deposition

- [x] Confirm that both raw and final processed data have been deposited in a public database such as GEO.
- [x] Confirm that you have deposited or provided access to graph files (e.g. BED files) for the called peaks.

Data access links<br>*May remain private before publication.*	ChIP-seq data have been deposited in NCBI under the BioProject accession number PRJNA771186. ChIP-seq files can be accessed from the following link: https://dataview.ncbi.nlm.nih.gov/object/PRJNA771186?reviewer=jklns645j96no59vkuh3clgkgf<br><br>Since NCBI sequence read archive (SRA) portal do not accept the BED files, we are exploring other options to deposit these files into GEO. Meanwhile, we have provided access to the BED files indicated below through our resource website (http://ecoli.med.utoronto.ca/eMap/TF).<br><br>1. Ynej ChIP_Tryptone_peaks.bed<br>2. Ynej ChIP_Putrescine_peaks.bed<br>3. Ydip ChIP_RM15_Ydip.bed<br>4. Ydip ChIP_MM15_Ydip.bed

Files in database submission	1. Library ID : 10_YdiP_FLAG_IP (Rich media at 15 degree)<br>Files : 10_YdiP_FLAG_IP_MAL13608_R1.fastq.gz (fastq)<br>10_YdiP_FLAG_IP_MAL13608_R2.fastq.gz (fastq)<br><br>2. Library ID : 12_YdiP_FLAG_IP (Minimal media at 15 degree)<br>Files : 12_YdiP_FLAG_IP_MAL13608_R1.fastq.gz (fastq)<br>12_YdiP_FLAG_IP_MAL13608_R2.fastq.gz (fastq)<br><br>3. Library ID : 6_YdiP_Mock (IgG only for YdiP in rich media at 15 degree)<br>Files : 6_YdiP_FLAG_Mock_R1.fastq.gz (fastq)<br>6_YdiP_FLAG_Mock_R2.fastq.gz (fastq)<br><br>4. Library ID : 8_YdiP_Mock (IgG only for YdiP in minimal media at 15 degree)<br>Files : 8_YdiP_FLAG_Mock_R1.fastq.gz (fastq)<br>8_YdiP_FLAG_Mock_R2.fastq.gz (fastq)<br><br>5. Library ID : 2_YdiP_Input (Whole chromatin for YdiP in rich media at 15 degree)<br>Files : 2_YdiP_FLAG_IP_R1.fastq.gz (fastq)<br>2_YdiP_FLAG_IP_R2.fastq.gz (fastq)<br><br>6. Library ID : 4_YdiP_Input (Whole chromatin for YdiP in minimal media at 15 degree)<br>Files : 4_YdiP_FLAG_IP_R1.fastq.gz (fastq)<br>4_YdiP_FLAG_IP_R2.fastq.gz (fastq)<br><br>7. Library ID : MAL9677_4_Input (Whole chromatin for YneJ in M9 + Putrescine)<br>Files : MAL9677_4_R1.fastq.gz (fastq)<br>MAL9677_4_R2.fastq.gz (fastq)<br><br>8. Library ID : MAL9677_5_Mock (IgG only for YneJ in M9 + Putrescine)<br>Files : MAL9677_5_R1.fastq.gz (fastq)<br>MAL9677_5_R2.fastq.gz (fastq)<br><br>9. Library ID : MAL9677_6_YneJ_Flag_IP (M9 + Putrescine)<br>Files : MAL9677_6_R1.fastq.gz (fastq)<br>MAL9677_6_R2.fastq.gz (fastq)<br><br>10. Library ID : MAL9677_7_Input (Whole chromatin for YneJ in M9 + Tryptone)<br>Files : MAL9677_7_R1.fastq.gz (fastq)<br>MAL9677_7_R2.fastq.gz (fastq)<br><br>11. Library ID : MAL9677_8_Mock (IgG only for YneJ in M9 + Tryptone)<br>Files : MAL9677_8_R1.fastq.gz (fastq)<br>MAL9677_8_R2.fastq.gz (fastq)<br><br>12. Library ID : MAL9677_9_YneJ_Flag_IP (M9 + Tryptone)<br>Files : MAL9677_9_R1.fastq.gz (fastq)<br>MAL9677_9_R2.fastq.gz (fastq)
Genome browser session<br>(e.g. UCSC)	Not applicable

Methodology

Replicates	We have performed 12 ChIP-seq experiments for two orphan genes (YneJ, YdiP) along with input (whole chromatin only) and mock (IgG only) experiments only once. After the FDR and p-value cut-off, the significant binding regions of YneJ and YdiP from the peak calling analysis are shown in Supplementary Data 8 and 12, respectively. From the ChIP-seq datasets, we tested two genes for YneJ and YdiP and reproduced the ChIP-seq results using ChIP-qPCR with three independent biological replicate experiments. These results are included in the Figures 4 and 6.
Sequencing depth	ChIP-seq libraries were created and sequenced on a single lane of an Illumina Hi-Seq 2500 (paired-end reads, 2 x 125 bp) at TCAG (The Centre for Applied Genomics) Toronto Hospital for Sick Children sequencing facility. The sequence depth for each experiment, along with the total number of reads, uniquely mapped reads, length of reads, and paired or single-end are shown below:<br><br>[1] Library ID: 10_YdiP_FLAG_IP (Rich media at 15 degree)<br>1. Sequencing depth: 97.1359128017542<br>2. Total number of reads (million): 3.58195<br>3. Uniquely mapped reads (million): 0.771794<br>4. Length of Reads (bp): 126

5. Paired or Single: Paired

[2] Library ID: 12_YdiP_FLAG_IP (Minimal media at 15 degree)
1. Sequencing depth: 314.572563475877
2. Total number of reads (million): 11.600068
3. Uniquely mapped reads (million): 1.775108
4. Length of Reads (bp): 126
5. Paired or Single: Paired

[3] Library ID: 6_YdiP_Mock (IgG only for YdiP in rich media at 15 degree)
1. Sequencing depth: 1417.79133
2. Total number of reads (million): 52.281978
3. Uniquely mapped reads (million): 3.034906
4. Length of Reads (bp): 126
5. Paired or Single: Paired

[4] Library ID: 8_YdiP_Mock (IgG only for YdiP in minimal media at 15 degree)
1. Sequencing depth: 1400.442864
2. Total number of reads (million): 51.642242
3. Uniquely mapped reads (million): 5.22565
4. Length of Reads (bp): 126
5. Paired or Single: Paired

[5] Library ID: 2_YdiP_Input (Whole chromatin for YdiP in rich media at 15 degree)
1. Sequencing depth: 1118.75622835389
2. Total number of reads (million): 41.254864
3. Uniquely mapped reads (million): 2.074498
4. Length of Reads (bp): 126
5. Paired or Single: Paired

[6] Library ID: 4_YdiP_Input (Whole chromatin for YdiP in minimal media at 15 degree)
1. Sequencing depth: 1119.0262168093
2. Total number of reads (million): 41.26482
3. Uniquely mapped reads (million): 4.333978
4. Length of Reads (bp): 126
5. Paired or Single: Paired

[7] Library ID: MAL9677_4_Input (Whole chromatin for YneJ in M9 + Putrescine)
1. Sequencing depth: 1434.54661440465
2. Total number of reads (million): 52.89984
3. Uniquely mapped reads (million): 46.97472
4. Length of Reads (bp): 126
5. Paired or Single: Paired

[8] Library ID: MAL9677_5_Mock (IgG only for YneJ in M9 + Putrescine)
1. Sequencing depth: 1509.331518
2. Total number of reads (million): 55.657582
3. Uniquely mapped reads (million): 48.826712
4. Length of Reads (bp): 126
5. Paired or Single: Paired

[9] Library ID: MAL9677_6_YneJ_Flag_IP (M9 + Putrescine)
1. Sequencing depth: 2234.87787269614
2. Total number of reads (million): 82.412576
3. Uniquely mapped reads (million): 73.912378
4. Length of Reads (bp): 126
5. Paired or Single: Paired

[10] Library ID: MAL9677_7_Input (Whole chromatin for YneJ in M9 + Tryptone)
1. Sequencing depth: 1486.74729313359
2. Total number of reads (million): 54.824774
3. Uniquely mapped reads (million): 46.166696
4. Length of Reads (bp): 126
5. Paired or Single: Paired

[11] Library ID: MAL9677_8_Mock (IgG only for YneJ in M9 + Tryptone)
1. Sequencing depth: 1494.739233
2. Total number of reads (million): 55.119482

3. Uniquely mapped reads (million): 51.929334
4. Length of Reads (bp): 126
5. Paired or Single: Paired

[12] Library ID: MAL9677_9_YneJ_Flag_IP (M9 + Tryptone)
1. Sequencing depth: 1429.01749164717
2. Total number of reads (million): 52.6959499999999
3. Uniquely mapped reads (million): 43.97285
4. Length of Reads (bp): 126
5. Paired or Single: Paired

Antibodies

1. Monoclonal Anti-Flag M2 antibody (Mouse)
Supplier: Sigma
Cat#: F1804
Lot#: SLBN5629V
Experiment: ChIP-seq or ChIP-qPCR

2. Rabbit IgG Isotype Control
Supplier: Invitrogen
Cat#: 10500C
Lot#:TA262556
Experiment: ChIP-seq (Input)

3. IgG from mouse serum
Supplier: Sigma
Cat#: 15381
Lot#: SLBQ7097V
Experiment: ChIP-seq (Mock)

Peak calling parameters

The raw sequence reads (*.fastq) were mapped to the E. coli W3110 genome using bowtie2 software (Source: Langmead, Ben, et al. "Scaling read aligners to hundreds of threads on general-purpose processors." Bioinformatics 35.3, 2019, 421-432) with zero mismatch tolerance. The duplicated reads were removed, and then sorted using samtools (Source: Li, Heng, et al. "The sequence alignment/map format and SAMtools." Bioinformatics 25.16, 2009, 2078-2079).

The command line program and parameters used for read mapping is as follows:

```
bowtie2 -p 8 -N 0 -x ${Ecolie_Bowtie2_Index} \
 -1 ${Sample_R1.fastq.gz} \
 -2 ${Sample_R2.fastq.gz} \
 -S ${Sample}.sam
 echo "Converting to BAM ..."
 samtools view -bS ${Sample}.sam > ${Sample}.bam
 echo "Sort BAM ..."
 samtools sort ${Sample}.bam -o ${Sample}_sorted.bam
 echo "Remove Dup ..."
 samtools rmdup ${Sample}_sorted.bam ${Sample}_sorted.nodup.bam
 echo "Index BAM ..."
 samtools index ${Sample}_sorted.nodup.bam
```

The resulting mapped reads (*.bam files) were then subjected to the Bioconductor package, MOSAiCS (MOdel-based one and two Sample Analysis and Inference for ChIP-Seq; Source: Pei Fen Kuan, Dongjun Chung, Guangjin Pan, James A Thomson, et al., "A Statistical Framework for the Analysis of ChIP-Seq Data" Journal of the American Statistical Association, 2009), for peak calling.

We used constructBin function generated binarized format from bam files using the following command line:

```
constructBins(infile = ${BAM file name},
        fileFormat = "bam",
        outfileLoc =${Directory to store bin files},
        byChr = FALSE,
        useChrfile = FALSE,
        chrfile = NULL,
        excludeChr = NULL,
        PET = FALSE,
        fragLen = 100,
        binSize = 20,
        capping = 0)
```

Data quality

To determine the peaks at FDR 5%, the binarized files were fitted into the MOSAiCS models using the following function:

```
mosaicsPeak(fitTFBS,
            signalModel = "2S",
            FDR = 0.05,
            maxgap = 100,
            minsize = 20,
            thres = 10)
```

The peak calling quality control was then achieved by cross-correlation analysis by identifying enriched regions with False Discovery Rate (FDR) and filtration steps from control (i.e., 'input' and 'mock' ChIP with immunoglobulin G antibody) ChIP-seq experiments performed in respective growth conditions. After employing FDR cut-off at 5%, the number of peaks identified in the ChIP-seq experiment is indicated below:

Library ID: 10_YdiP_FLAG_IP (Rich media at 15 degree): 143 peaks
Library ID: 12_YdiP_FLAG_IP (Minimal media at 15 degree): 54 peaks
Library ID: MAL9677_6_YneJ_Flag_IP (M9 + Putrescine): 1,575 peaks
Library ID: MAL9677_9_YneJ_Flag_IP (M9 + Tryptone): 1,262 peaks

Next, the MOSCAiCS algorithm with a Hidden Markov statistical Model (HMM) was implemented, which provided a model-based approach for modeling read counts in ChIP-seq experiments and distinguished enriched reads from background noise (Source: Chung, Dongjun, Qi Zhang, and Sündüz Keleş. "MOSAiCS-HMM: A model-based approach for detecting regions of histone modifications from ChIP-seq data." Statistical Analysis of Next Generation Sequencing Data. Springer, Cham, 2014. 277-295). Notably, the MOSCAiCS-HMM determined the p-value significance of each peak from the fitted model (i.e., smaller the p-value, higher the probability of the peak being genuine). Peaks with p-value ≤ 1 x 10-4, and genes with start codons within 200 bp upstream and downstream of a binding site for YneJ, and genes with start codons from 50 bp upstream and downstream of a binding site for YdiP was considered as potential YneJ or YdiP targets. The resulting binding sites after employing these stringent filtering parameters, along with their p-values, are shown in Supplementary Data 8 and 12.

Software

The raw ChIP-sequence reads were mapped to the E. coli W3110 chromosome using bowtie2 open-source software. After removing duplicates, the uniquely mapped reads sorted by samtools were subjected to Bioconductor MOSAiCS (MOdel-based one and two Sample Analysis and Inference for ChIP-Seq) package for peak calling. Artifactual regions were excluded from further analysis if the peaks called in the ChIP-seq experiments were present in input and/or mock experimentations.
