# Supplementary material for: Auxotrophic and prototrophic conditional genetic networks reveal the rewiring of transcription factors in Escherichia coli
Source: Nat Commun. 2022 Jul 14;13:4085. doi: 10.1038/s41467-022-31819-x (PMC9283627; doi:10.1038/s41467-022-31819-x)
Supplement: Supplementary file 3 — Description of Additional Supplementary Files [file 41467_2022_31819_MOESM3_ESM.pdf]

Title: Supplementary Data 1.
Description: TF genes targeted for genetic screening.

Title: Supplementary Data 2.
Description: TF gene pairs with significant GI scores in static networks.

Title: Supplementary Data 3.
Description: Functional crosstalk between enriched bioprocesses in RM network.

Title: Supplementary Data 4.
Description: Correlation score of GI gene pairs in static (RM, MM) and differential (DF) networks.

Title: Supplementary Data 5.
Description: Bioprocesses enriched with GIs involving global or local TF regulators.

Title: Supplementary Data 6.
Description: Number of GIs identified per orphan TF.

Title: Supplementary Data 7.
Description: Inter-connectivity of orphan TFs with annotated genes in enriched bioprocesses.

Title: Supplementary Data 8.
Description: YneJ binding sites under carbon-limiting conditions by ChIPseq.

Title: Supplementary Data 9.
Description: Gene pairs with differential (DF) GI scores.

Title: Supplementary Data 10.
Description: Autocorrelated TF genes and their corresponding single gene deletion mutant strain fitness from phenotypic genetic screen (Tong et al., 2020).

Title: Supplementary Data 11.
Description: TF gene pairs connecting distinct modules in differential network.

Title: Supplementary Data 12.
Description: YdiP binding sites under carbon-limiting conditions by ChIPseq.

Title: Supplementary Data 13.
Phylogenetic profiles of each TF target across 4,409 species in 17 bacterial phyla.

Title: Supplementary Data 14.
Description: Conserved TFs with epistatic connections from static and differential (DF) networks in each of the assigned bioprocesses.

Title: Supplementary Data 15.
Description: Co-conserved interprocess gene pairs connected by GIs in static and differential (DF) networks.

Title: Supplementary Data 16.
Description: Protein-BLAST searches to determine putative TF paralogs.

Title: Supplementary Data 17.
Description: Bacterial strains, oligonucleotides, and antibodies used in this study.
